# *Epormenis cestri* secretions in *Sebastiania schottiana* trees cause mass death of honey bee *Apis mellifera* larvae in Uruguay

**DOI:** 10.1371/journal.pone.0190697

**Published:** 2018-01-09

**Authors:** Ciro Invernizzi, Enrique Nogueira, Pablo Juri, Estela Santos, Daniela Arredondo, Belén Branchiccela, Yamandú Mendoza, Karina Antúnez

**Affiliations:** 1 Sección Etología, Instituto de Biología, Facultad de Ciencias, Montevideo, Uruguay; 2 Instituto de Producción Animal, Facultad de Veterinaria, Montevideo, Uruguay; 3 Laboratorio de Microbiología, Instituto de Investigaciones Biológicas Clemente Estable, Montevideo, Uruguay; 4 Laboratorio de Apicultura, Instituto de Investigación Agropecuaria, Colonia, Uruguay; Philipps-Universitat Marburg Fachbereich Biologie, GERMANY

## Abstract

For more than 60 years, sporadic cases of massive summer honey bee larvae mortality in colonies located near freshwater systems with abundant riparian vegetation have been reported in Uruguay. This odd phenomenon, known as “River disease” by beekeepers, can lead to colony death by depopulation. The aim of this study was to detect the causes of larvae death. Different experiments and analyses were performed using affected apiaries located between two important water courses. 1 day old larvae were the most susceptible and substances that killed the larvae were present in the nectar but not in the pollen. A palynological analysis of nectar samples showed that bees collect this resource from commonly pollinated floral species in the country. However, abundant fungi spores and conidia were found, which indicates that the bees also collected honeydews. In the riparian vegetation, bees were observed collecting the secretions of the planthopper *Epormenis cestri* on *Sebastiania schottiana* trees. It was found that the mortality period of larvae overlaps with the presence of *E*. *cestri*. Larvae maintained in the laboratory were fed (i) nectar from healthy colonies, (ii) nectar from affected colonies, and (iii) secretions of *E*. *cestri*. The mortality of the larvae that received nectar from colonies affected with River disease and secretions of *E*. *cestri* was higher than the mortality of those receiving nectar from healthy colonies. This represents the first report of planthopper honeydew causing mass larval mortality in honey bees.

## Introduction

Beekeepers in Uruguay call the mass larval mortality that occurs in late spring and early summer in colonies located close to freshwater systems with abundant riparian vegetation “River disease”. The main symptom of affected colonies is the partial or total absence of larvae. First, a spotted brood pattern or even a total loss of larvae (in the severe cases) is observed. After a few days only eggs are present. These colonies usually have plenty of honey and pollen stored, probably because there are no larvae to feed. Over time, the colonies begin to depopulate due to the lack of replacement of dead bees and if this situation is not reverted, significant colony losses can occur in late summer and autumn [1–2]. River disease was first mentioned in 1951 by a newspaper that reported it in colonies located on the edge of the Santa Lucia river (southern Uruguay), but the disease remained practically unknown among beekeepers through the following decades. However, in the last few years, the River disease has been frequently reported in colonies located close to rivers and streams in the basins of the Uruguay, Negro and Cuareim Rivers (western Uruguay). Economic losses caused by this disease are significant; it is an important contributor to honey production decline and increases colony mortality [2].

Although River disease is not observed annually, it can occur during several successive years in a particular area. No practical measures have been found to deal with this disease. Some beekeepers have tried unsuccessfully to reduce larvae losses by supplying sugar syrup, causing beekeepers to remove their colonies from the affected areas when the larvae begin to die.

The cause of River disease has not been identified. The most common brood diseases in honey bees have been discarded. Experiments performed in the 1970s by technicians from the Ministry of Livestock, Agriculture and Fisheries suggest an environmental origin [1]. Colonies with River disease have managed to recover very slowly without treatment. When adult bees from an affected colony were transferred to a colony with healthy brood and food, the colony developed without problems. However, when adult bees from a healthy colony were transferred to a colony with affected brood and food, larval survival was very low. Moreover, a comb with eggs taken from a colony with River disease and put in a healthy colony resulted in healthy larvae [2]. These results have been validated by beekeepers, who found that by placing colonies affected by River disease on a new site, larval mortality begins to decline and colonies recover.

Among the environmental causes that could explain larvae death has been proposed the shortage of food in spring [3] or a toxic substance present in pollen [4].

Honey bees are exposed to a variety of toxic substances, both botanical and anthropogenic in origin, in their foraging environment [5]. Some specialist insects have evolved specific mechanisms to counteract the effects of toxic substances present in certain nectars and/or pollens [6]. However, a generalist species such as *Apis mellifera* may be susceptible to some of the substances that plants produce to defend themselves from predators [7–9], although recently the beneficial role of some secondary metabolites in pollinators has been highlighted [10].

Substances that kill larvae in colonies with River disease could enter the hive with the products that foragers collect: nectar, honeydew, pollen, water and resins. Water and resins can hardly be the route of entry of toxic substances. Honey bees collect water from diverse sites such as dewdrops or tree holes, then it doesn’t seems to be very likely for a toxic substance to enter the hive trough that route. Resins are also unlikely to be involved because of its composition, function and the amount that enters the hives [11]. Additionally, if water or resins were responsible for the larvae dead, their effects should not be restricted to a short period of the year. Thus, nectar, honeydew or pollen must be the source of substances that kill larvae. Nectar and pollen of a large number of plant species may affect larvae and/or adult bees [9]. The most similar case to River disease occurs in California (USA) with California Buckeye (*Aesculus californica*) whose nectar and pollen are toxic to both larvae and adult bees. In colonies that forage *A*. *californica* shrubs, eggs do not hatch or larvae die within three days after hatching and are rapidly removed by adult bees [12]. Unlike this case, River disease does not affect adults [1–2].

About honeydew, bees usually collect the sweet secretions of insects when they are abundant [13]. In Uruguay, the native planthopper *Epormenis cestri* (Hemiptera, Flatidae) is frequently found in several tree species of riparian vegetation with visible secretions [14].

This study aims to determine the viability of embryos and larvae in colonies affected by River disease and to identify the primary cause of the problem.

## Materials and methods

This study was performed from January to February 2015 and December 2015 to February 2016. In January 2015, River disease was reported in several apiaries located between the Yi River and the Maciel stream in the department of Durazno (Uruguayan center region). One affected fixed apiary was selected (Apiary 1), located 1.6 km to Yi River and 2.3 km to Maciel stream. Samples of nectar and pollen from colonies that had total loss of larvae were taken. Experiments were conducted to determine first, how the problem affected embryos and larvae, and second, the role of nectar and pollen in larvae death. The mean temperature during this first period was 24.4°C (range: 19.9–29.6°C).

The following summer, the work started immediately after the first symptoms of the disease were observed in the same apiaries (early December, 2015). Another fixed apiary was selected (Apiary 2), located at 1.8 km to Yi River and 1.8 km to Maciel stream. Samples of nectar and pollen from affected colonies (total loss of larvae) were taken. The riverine vegetation of Yi River and Maciel stream was carefully studied in order to identify the resources collected by bees. Bees were found collecting secretions of the planthopper *Epormenis cestri* (Berg, 1879) in *Sebastiania schottiana* trees (Müll. Arg.) Müll. Arg. From mid-December until the end of February, the presence of nymphs and adults of *E*. *cestri* and bees collecting their secretions were recorded. In vitro larval rearing experiments were performed to determine if the *E*. *cestri* secretions on *S*. *schottiana* trees were causing larval death. The mean temperature during this second period was 26.1°C (range: 18.5–30.1°C).

All the colonies used in this study were regularly inspected in order to verify the presence of the queen.

The study was carried out on private land; the owner of the land gave permission to conduct the study on this site. The field studies did not involve endangered or protected species.

### Embryo and larvae mortality

As it is very difficult to find larvae in River disease affected colonies, a possible explanation may be the inviability of embryos. To determine whether River disease affects the survival of embryos (eggs losses), 6 frames with eggs were taken from three affected colonies from Apiary 2 and placed in two healthy colonies (three frames per colony) in an unaffected apiary. Combs were photographed before the introduction in the new colonies and 9 days later, using a specially designed device to hold the camera (Nikon 5200 SLR with a 50mm lens) and the frame. The survival rate of eggs was determined by analyzing 1000 total cells per colony.

To determine how River disease affects the survival of larvae of different ages, two combs with eggs and larvae of all ages obtained from healthy colonies located in an unaffected apiary, were added to three affected colonies from Apiary 1 that only had eggs. Before placing them into the hives and for the next 5 days combs were photographed. The daily mortality of three days old eggs and of the 1–5 days old larvae was visually estimated from photographs.

### Botanical origin of nectar and pollen

Samples of nectar from uncapped cells and pollen (extracted from the surface of the cells) from colonies with River disease from Apiary 1 (9 colonies that had been sick for at least 20 days) and Apiary 2 (9 colonies sampled within 10 days of initial larvae deaths) were analyzed.

The botanical origin of the nectar and pollen samples was determined by observing 600 pollen grains under a microscope (400X) and identified by comparing them to a reference collection [15]. Groups of fungi and conidia from nectar samples were identified using reference images [16–17].

To determine if *E*. *cestri* secretions left any signal that allows its identification in nectar samples, the cottony white material that surrounds the nymphs was observed under the microscope. To do this, *E*. *cestri* nymphs were washed with distilled water in an Eppendorf tube and a drop of liquid was observed at 400X.

### Role of nectar and pollen in larvae mortality

Three mesh tents (6 x 4 m) were installed at the National Agricultural Research Institute—La Estanzuela (Colonia, Uruguay), and 4 small colonies (6000–8000 bees) without reserves of pollen or nectar (a comb with brood, two empty combs and an internal feeder) were placed in each tent. The colonies were fed *ad libitum* with different combinations of pollen (cakes) and nectar extracted from healthy colonies and colonies affected by River disease (Apiary 1): i) pollen and nectar from healthy colonies, ii) pollen from affected colonies and nectar from healthy colonies, iii) pollen from healthy colonies and nectar from affected colonies, and iv) pollen and nectar from affected colonies. Three colonies (one colony per tent) were assigned to each combination. After three days, each comb with brood was replaced by a new previously photographed comb with eggs. These combs were removed from each colony at day 9 and photographed again. The percentage of viable offspring was determined by counting the number of eggs that continued their development until pupae stage.

### Bees that collect the secretions of *Epormenis cestri* in the trees *Sebastiania schottiana*

On December 10^th^ 2015 the total absence of larvae in 9 colonies in the Apiary 2 was verified. These colonies had been inspected 10 days before without detecting larvae losses. The presence of larvae in newly sealed cells indicated that the mortality of larvae had started suddenly, approximately on December 4^th^.

From December 13 to January 5, a section of the Maciel stream riparian vegetation was examined 6 times, counting and discriminating between nymphs and adults 100 specimens of *E*. *cestri* per tree, in 6 *S*. *schottiana* marked trees. The presence of bees collecting *E*. *cestri* secretions in the same trees was recorded by walking at a steady pace (20 seconds per tree approximately) counting the bees, three times a day (9.00, 12.00 and 16.00 hours). This was done 11 times between December 23 and February 18.

Finally secretion samples of *E*. *cestri* present in the leaves of *S*. *schottiana* were taken with a little spatula and kept at -20°C for further use in larval rearing experiments.

### Determination of the period in which colonies can contract River disease

To determine the moment the food resources causing larvae mortality no longer entered the colony, healthy colonies were relocated to the Apiary 1 on December 30 (10 colonies), January 18 (10 colonies), January 30 (4 colonies) and February 16 (6 colonies). On February 18 in order to discard the possibility that new colonies contracted River disease by robbing honey from affected and depopulated colonies, 4 colonies were located to a nearby apiary from the apiary 1, strongly affected by River disease and where only two colonies were not removed.

### Role of *Epormenis cestri* secretions on bee larvae mortality

One day old larvae from a healthy colony were extracted from the cells and placed in 96 plastic well microplates. The larvae continued their development in the laboratory under conditions described by Evans [18]. Three replicates of five groups of 24 larvae each were used. Group 1 was fed with an artificial diet composed of 1/6 syrup, 1/6 nectar from healthy colonies and 4/6 of royal jelly; group 2 was fed with 1/6 syrup, 1/6 nectar from diseased colonies and 4/6 of royal jelly; group 3 was fed with 1/9 syrup, 1/9 nectar from healthy colonies, 1/9 secretions of *E*. *cestri* and 6/9 of royal jelly; groups 4 and group 5 (controls) were fed only with 1/3 syrup; 2/3 royal jelly. Nectar was carefully extracted from uncapped cells from healthy and River Disease affected colonies. Syrup was composed by 18% fructose, 18% glucose and 3% yeast extract. Larvae were inspected every day recording mortality. To show that larvae mortality was not caused by volatile substances, two different microplates were used: larvae from group 4 (control 1) were developed in the same microplate that group 1 while the larvae from group 5 (control 2) were on the same microplate that groups 2 and 3. After the first 48 hours, larvae were taken out of the incubator and examined daily for five days. Surviving larvae were transferred to new microplates filled with fresh artificial food of each treatment.

### Statistical analysis

The number of daily dead larvae from different ages located in affected colonies as well as the ones reared under laboratory conditions, was recorded in a data table. Survival analysis was performed with the Kaplan Meier method. The difference between the survival curves was calculated with the Log-Rank and Holm-Sidak test a posteriori. The program used for the analysis was SigmaStat 3.5 Software Jandel Scientific (San Jose, CA, USA).

Larvae mortality in tent confined colonies fed with different combinations of nectar and pollen from healthy colonies and affected by River disease was analyzed using the Kruskal-Wallis test. Mann-Whitney test was used to compare paired samples.

The presence of bees collecting *E*. *cestri* secretions in *S*. *schottiana* trees at different times was analyzed using Chi^2^ test with data from the first two records. The program used for the analysis was Past 2.09 Software.

## Results

### Embryo and larvae mortality

Only 6.2% and 4.4% of the eggs from colonies affected with River disease placed in two healthy colonies did not reach the pre-pupae stage.

**Fig 1:**
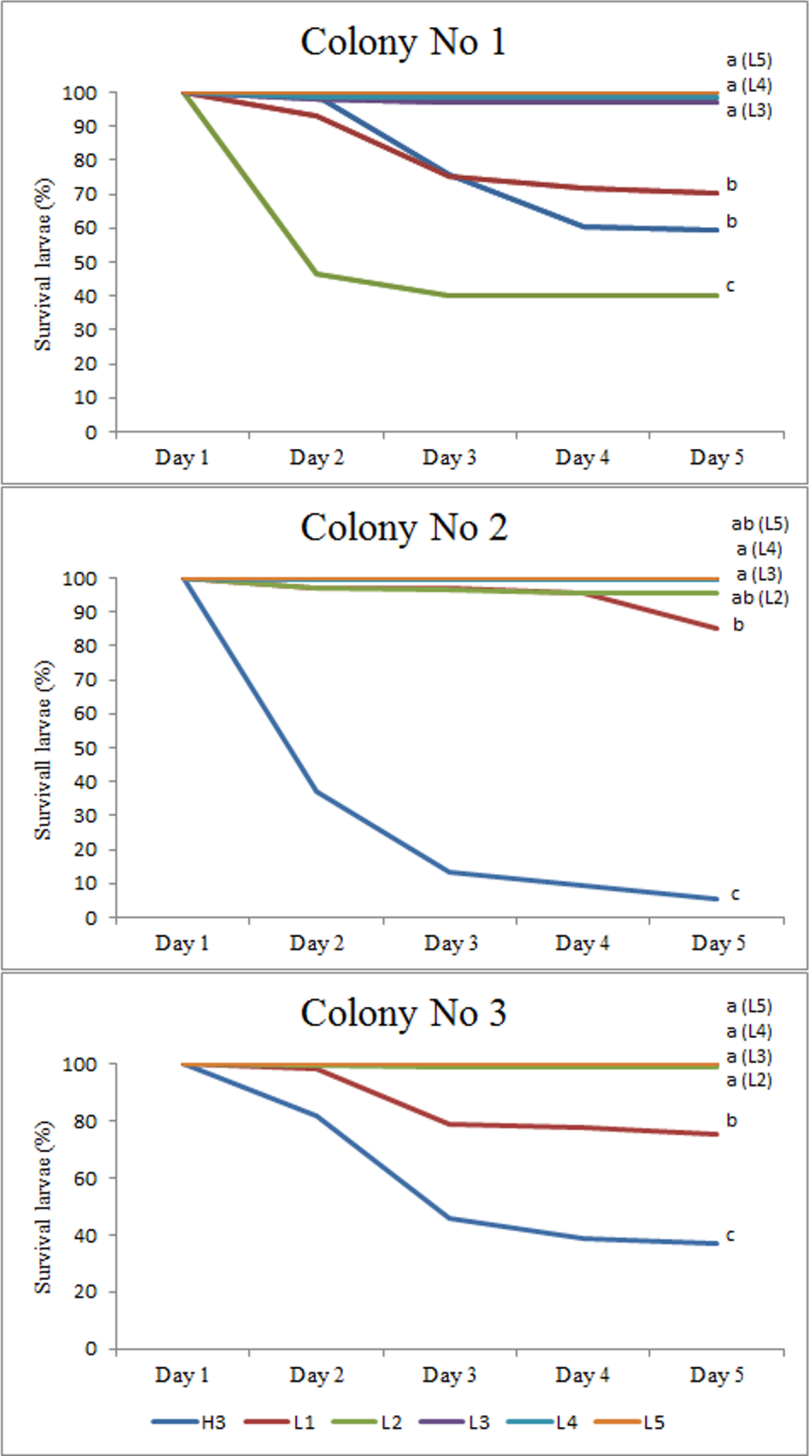
Larvae survival curves in colonies with River disease. Three days old eggs (H3) and 1 to 5 days old larvae (L1 to L5) were translocated from healthy colonies to three colonies with River disease for a period of five days. Different letters indicate significant differences (P <0.05) in larval survival (Holm Sidak test).

The stage most affected by River disease was determined using the 5 day sequence of comb photographs containing eggs and larvae of all ages in three affected colonies. The number of cells observed in the three colonies was 456, 709 and 623. Significant differences in survival curves between different age groups of larvae were found in the three colonies (Log-Rank test, Colony 1: 160.12, P <0.001; Colony 2: 708.28, P <0.001; Colony 3: 257.14, P <0.001) (Fig 1). Considering the three colonies pooled together: the youngest larvae (emerged from the eggs) had higher mortality (64.6%), particularly in Colony 2, followed by 2 days old larvae (28.5%) and 1 day old larvae (21.8%). The mortality of 2 days old larvae is due to the abnormally high mortality of larvae in Colony 1 (60%). Larvae groups 3, 4 and 5 days hardly suffered losses (Fig 1).

### Botanical origin of nectar and pollen

The palynological analysis of samples of nectar and pollen obtained from the two apiaries showed that bees collected food from commonly pollinated floral species. Common pollens to all nectar samples in Apiary 1, ordered according to their frequency, were *Lotus* spp., *Trifolium repens*, *Medicago sativa* and *Glycine max*, while in the Apiary 2 the native Myrtaceae, *Echium plantagineum*, *Eucalyptus* spp. and *Trifolium repens* were found. These pollens represented on average more than 85% of the pollen types found in the samples from each apiary. Pollens from other species were found at very low frequencies and not in every sample.

In all nectar samples of both apiaries, a small amount of pollen and abundant honeydew elements were found. Fungi spores and conidia corresponded mostly to *Gonadobotrium*, *Trichomerium foliicola*, *Tetracladium*, *Bipolaris*, *Drechslera*, *Alternaria* and *Cladosporium* types. Also, in all nectar samples of Apiary 2 fungi *Spegazzinia*, *Gonoderma* and *Metacapnodium spongiosum* type were found.

**Fig 2:**
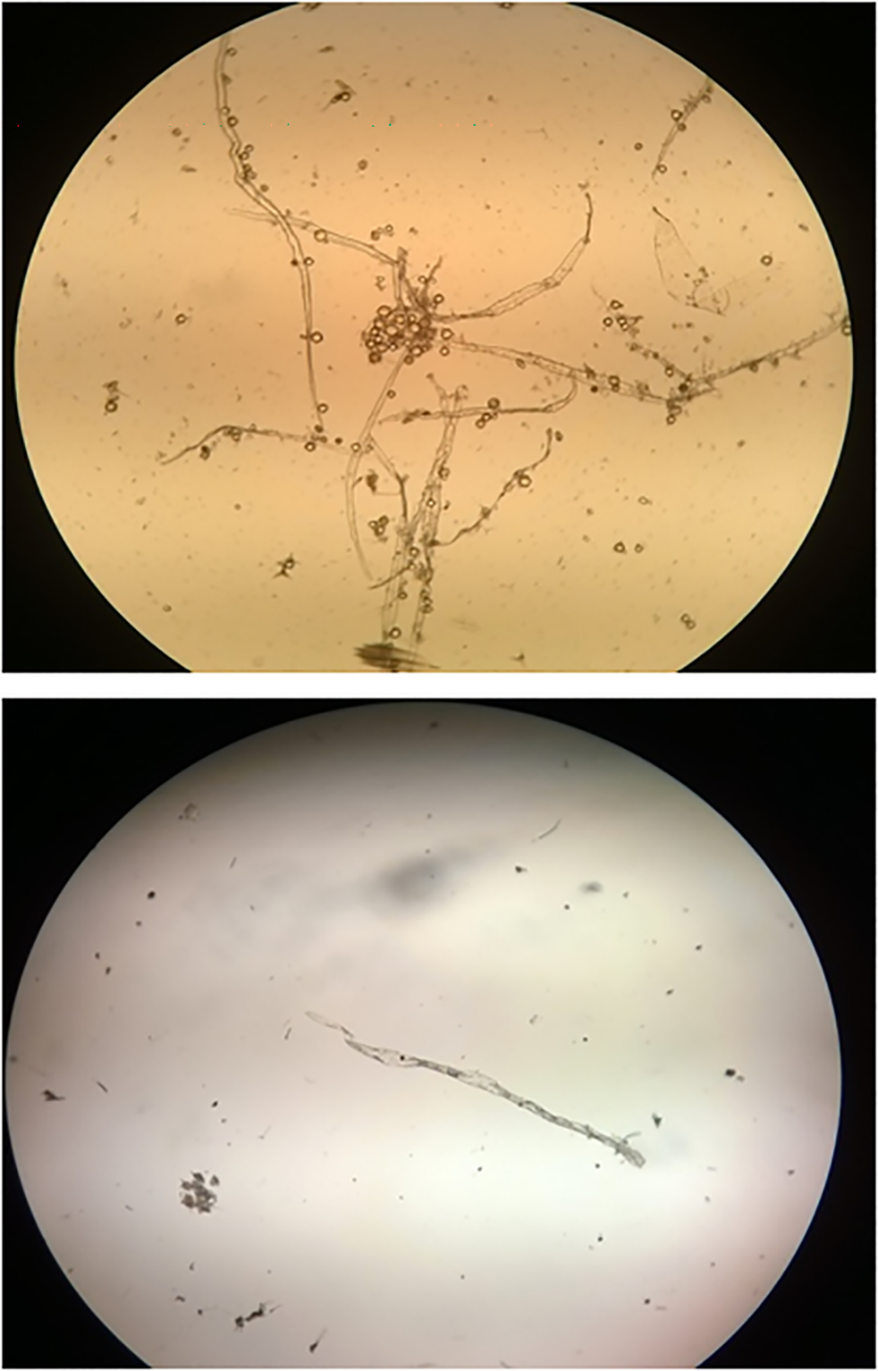
Fibers from the cottony cover of *E*. *cestri* nymphs. Fibers were found in nectar samples of colonies with River disease (above) and in the wash water of *Epormenis cestri* nymphs (below).

The microscopic analysis of nectar samples showed rare non-rigid fibers, identical to those observed in the water with which *E*. *cestri* nymphs were washed (Fig 2). The source of these fibers is the cottony material covering the nymphs.

Common pollens recorded from Apiary 1, ordered according to their frequency, were *Lotus* spp., *Medicago sativa* and *Trifolium repens*. These pollens represented on average over 70% of the pollen types found in the samples. Other species that appeared in low frequency (<2%) from Apiary 1 included *Cynara cardunculus*, *Baccharis trimera*, *Ludwigia peploiodes* (aquatic plant) and an unidentified species. Common pollens recorded from Apiary 2, ordered according to their frequency, were *Echium plantagineum*, native Myrtaceae *Eucalyptus* spp. and *Trifolium repens*. These pollens represented on average more than 85% of the pollen types found in the Apiary 2.

### Nectar and pollen role in larvae mortality

**Fig 3:**
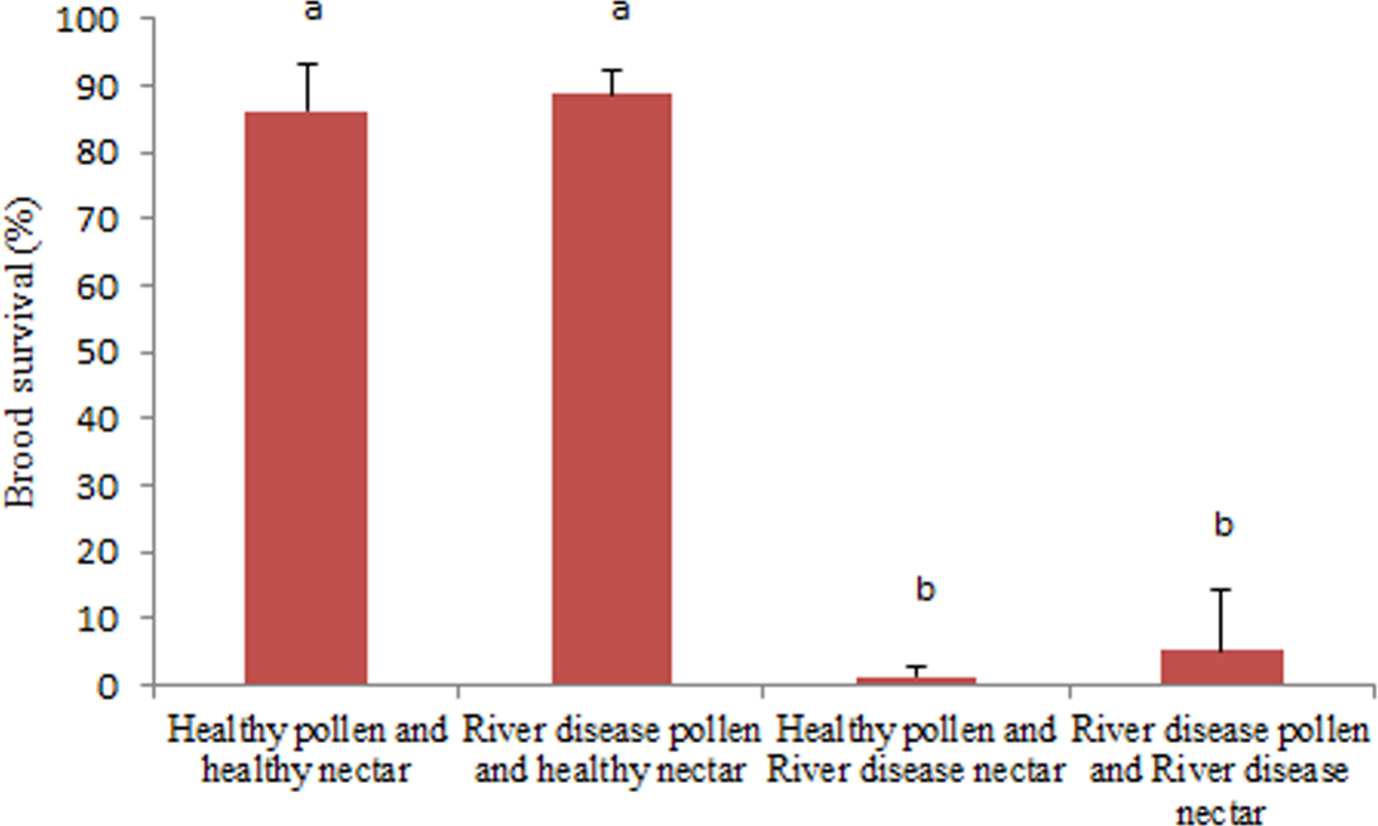
Nectar and pollen role in larvae death in colonies affected with River disease. Percentage of brood survival in colonies placed in tents and fed with different combinations of nectar and pollen obtained from healthy colonies and colonies affected with River disease. Different letters indicate significant differences (P <0.05) (Mann Whitney test).

Larvae mortality in colonies from the four nectar and pollen groups (from healthy and River disease affected colonies) exhibited significant difference. The two groups of colonies receiving nectar from colonies affected by River disease (groups iii and iv) suffered almost total loss of larvae, while the remaining colonies (groups i and ii) did not exceed 15% of larvae loss (H: 8.33; P = 0.04) (Fig 3).

### Bees that collect the secretions of *Epormenis cestri* in the trees *Sebastiania schottiana*

**Fig 4:**
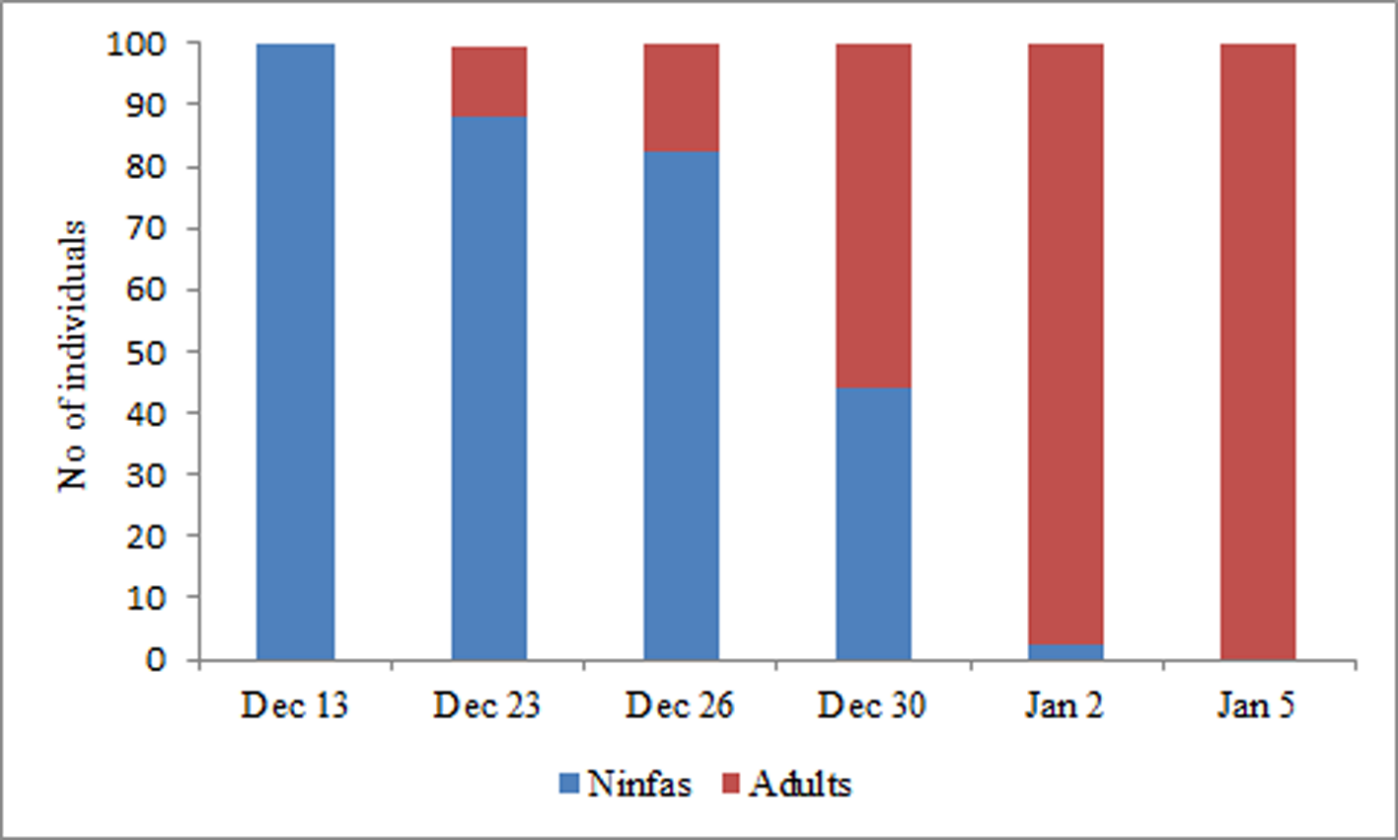
Proportion of *Epormenis cestri* nymphs and adults (100 individuals) observed in *Sebatiania schottiana* trees.

The proportion of nymphs and adults of *E*. *cestri* in *S*. *schottiana* was registered 6 times between December 13 and January 5. The number of *E*. *cestri* nymphs declined as they molted into adults and therefore in the first survey only nymphs were found, whereas in the last one only adults were detected (Fig 4). The total number of *E*. *cestri* gradually declined since the last record and on February 12 almost no specimens or their secretions on leaves of *S*. *Schottiana* were observed.

**Fig 5:**
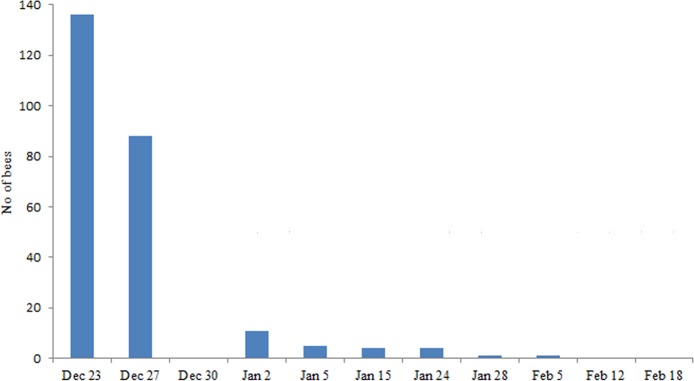
Total number of bees observed foraging *Epormenis cestri* secretions on *Sebastiania schottiana* trees. Recordings were made in three trees, three times a day (9.00, 12.00 and 16.00 hours).

The presence of bees collecting *E*. *cestri* secretions was recorded 11 times from December 23 to February 18 at 9.00, 12.00 and 16.00 hours. The number of foraging bees decreased 36% between the first record and the second one carried out four days later, and then fell sharply during January (December 30 no bees were observed due to rain). By mid-February no bees on *S*. *schottiana* were recorded (Fig 5).

The first two records showed no differences in the number of bees at the three moments of the day (December 23: Chi ^2^ = 1.44; P = 0.49; December 27: Chi2 = 1.12; P = 0.57).

### Period in which colonies can contract River disease

Healthy colonies moved to Apiary 1 on December 30 (10 colonies), January 18 (10 colonies), January 30 (4 colonies) had total loss of larvae within 10 days of their installation. In contrast, 2 of the 6 colonies moved to the apiary on February 16 did not develop River disease. Four colonies transferred on February 18 to an apiary near Apiary 1 (where most of the colonies affected by River disease had been removed) had no larvae loss.

### Role of *Epormenis cestri* secretions on bee larvae mortality

**Fig 6:**
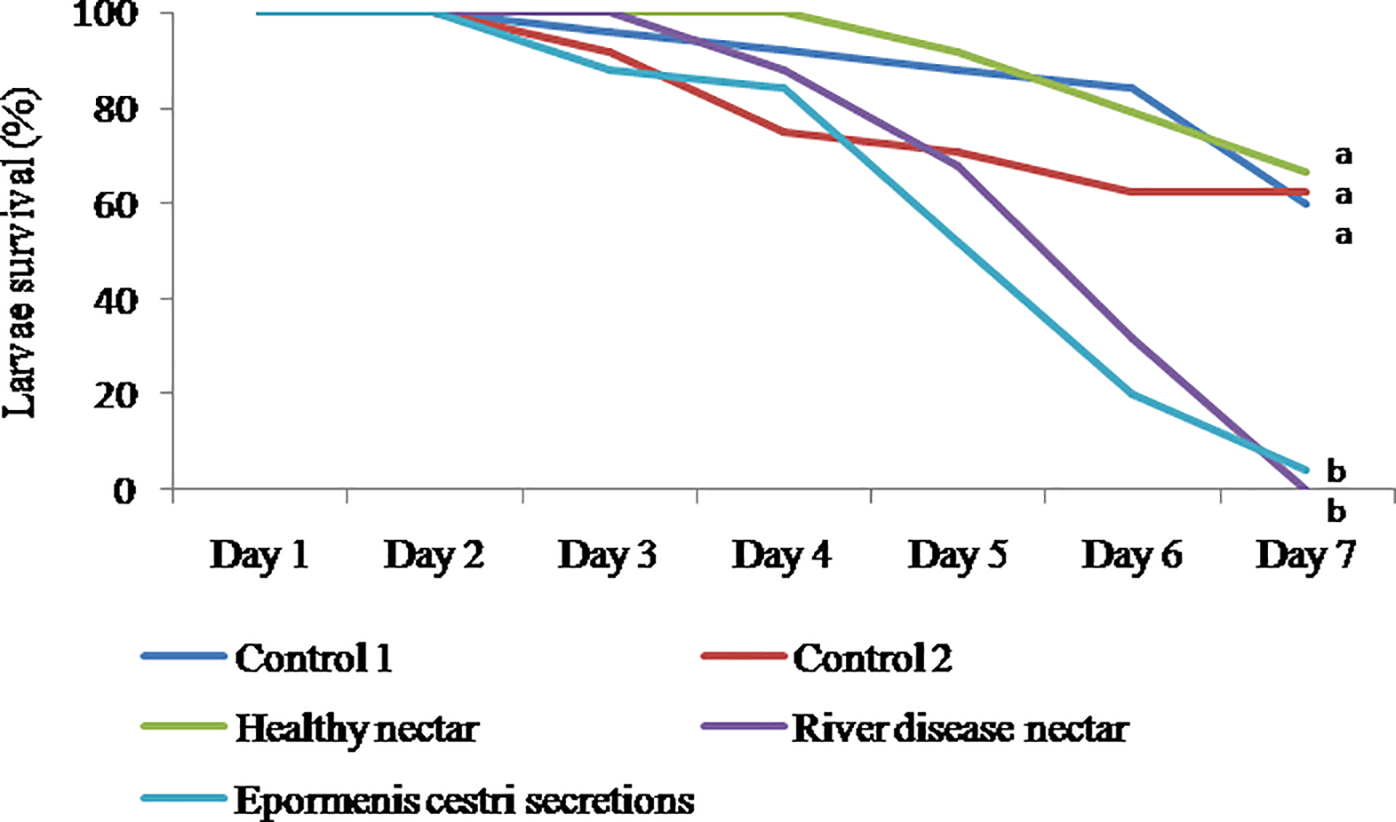
Survival curves of laboratory reared larvae fed with different diets. Control 1: larvae were fed with 1/3 syrup and 2/3 royal jelly; Control 2: larvae were fed with 1/3 syrup and 2/3 royal jelly; Healthy nectar: larvae were fed with 1/6 syrup, 1/6 nectar from healthy colonies and 4/6 of royal jelly; River disease nectar: larvae were fed with 1/6 syrup, 1/6 nectar from colonies with River disease and 4/6 of royal jelly; *Epormenis cestri* secretions: larvae were fed with 1/9 syrup, 1/9 nectar from healthy colonies, 1/9 secretions of *E*. *cestri* and 6/9 of royal jelly. Different letters indicate significant differences (P <0.05) in larval survival (Holm Sidak test).

The mortality of larvae over 7 days varied according to the feeding treatment (Log-Rank test: 52.92; P <0.001). Larvae fed with nectar from healthy colonies (Group 1) or syrup (Groups 4 and 5) presented a mortality of 40%, 37% and 33% respectively, at the end of the period. However, larvae receiving nectar from colonies affected by River disease (Group 2) or secretions of *E*. *cestri* (Group 3) presented a mortality of 100% and 96%, respectively (Fig 6).

## Discussion

River disease was reported over 60 years ago in Uruguay and has had devastating effects on the areas it occurs. Although specific experiences and observations from beekeepers indicated that it had an environmental origin [1–2], it has never been fully characterized and its causes were still unknown. The set of analysis and assays carried out in this study characterize and identify the cause of River disease.

The translocation of combs with eggs from colonies affected with River disease to healthy colonies demonstrated that the disease does not affect the embryonic development of bees. Almost 95% of the eggs developed into pre-pupae, a value within the normal range for healthy colonies [19]. This result is consistent with findings from the first descriptions of the disease [1].

The daily monitoring of translocated combs from healthy colonies to colonies affected with River disease showed that larvae within 24 hours from hatching (from three days old eggs present in the combs) were the most affected with 65% mortality. 1 to 2 days old larvae had reduced mortalities (less than half). This observation of the highest mortality in the youngest larvae had been previously observed in sick colonies [1–2]. The reason why losses in 1–2 days old larvae was not higher (in resident colonies with stationary combs had no surviving larvae) could be due to those larvae being fed by nurses from their healthy colony previous to their introduction into colonies affected with River disease. This did not occur when the larvae emerge from the eggs, which were fed exclusively by nurse bees of diseased colonies. Nevertheless, the loss of these early-stage larvae was not total as expected. Larvae aged 3, 4 and 5 days suffered low losses, indicating that late-stage larvae are not affected by River disease.

The palynological analysis of nectar and pollen samples showed that bees in this study foraged from commonly pollinated floral species in Uruguay [20–21]. This result indicates that larvae mortality cannot be attributed to nectar or pollen from the plant species found. However, a small amount of pollen and a large number of fungi and conidia spores of different groups was found in the nectar, indicating the presence of honeydew [16–17].

At the beginning of this study, pollen seemed as a probable cause of larvae deaths because of the toxic substances present in several plants [3–6]. Also, there are examples of larvae deaths caused by the pollen of *Aesculus californica* in USA and *Stryphondenddron polyphyllum* in Brasil [12–22]. However, experiences with colonies confined in tents were decisive to identify nectar (with honeydew) and discard pollen, as the only food that causes mortality of larvae.

The observation of many bees foraging *E*. *cestri* secretions on leaves of *S*. *schottiana* would explain the abundance of elements associated to honeydew found in the nectar. The fact that the fibers found in the nectar were also observed in the water which the planthopper nymphs were washed, confirms the origin of the honeydew collected by the colonies affected with River disease. Thus, secretions of *E*. *cestri* were considered as the possible cause of larvae death.

*E*. *cestri* nymphs were found after the abrupt loss of larvae in colonies from Apiary 2, *E*. *cestri* nymphs were found molting into adults for the next 20 days during which the colony population begins to decrease. Both nymphs and adults were concentrated in the branches of *S*. *schottiana* secreting copious amounts of sugary liquids falling on the leaves. The number of bees collecting secretions declined rapidly despite the abundant secretions visible in *S*. *schottiana* leaves. Very few bees were found 30 days after larvae mortality started in the area. This decline could be due to two causes. First, colonies were able to replace dead bees for just 20 days after larvae mortality started, resulting in a decreased number of foragers. Second, the lack of brood may reduce the incentive for the bees to collect food [23]. This decrease in foraging activity was clearly observed when the activity around the entrance of affected colonies (including the well-populated colonies) was compared with colonies that entered the apiary at different times.

Colonies introduced to the apiary on four occasions between December 30^th^ and February 16^th^ contracted River disease within a few days, except for the last group where two of the six colonies did not show evidence of dead larvae. It is very likely that in the other four colonies, larval death was due to honey robbing from the weakened or dead neighbor colonies affected by River disease. Evidence in support of this hypothesis includes, two days later, four colonies were taken to a nearby apiary where there was only one healthy colony and one affected with River disease, none presented larvae mortality. Given that on February 12^th^ there were very few specimens of *E*. *cestri* and secretions were no longer seen, these results strongly suggest that *E*. *cestri* secretions are involved with larvae mortality.

Larvae reared in the laboratory showed that almost all the larvae that were fed with River disease nectar or *E*. *cestri* secretions (only 11% of all food received) died over 7 days while less than 40% of the larvae fed with fructose or nectar from healthy colonies died. This experience, while indicating that the secretions of *E*. *cestri* are responsible for the differential larvae death, does not reproduce the same conditions in which larvae in a colony affected with River disease die. In laboratory conditions, most of the larvae die after the fifth day; while in an affected colony almost all the larvae die within the first 24 hours (it is extremely difficult to find larvae in very sick colonies). This remarkable difference can be explained if the amount of secretions of *E*. *cestri* supplied to the larvae directly or as part of nectar during the experiment was at a lower proportion than what larvae receive in natural colonies. It must be taken into consideration that in their first hours larvae receive food in surplus and are fed as necessary only after the second day [24]. These differences in larval feeding may explain why in natural colonies larvae die in the first hours. Another possibility is that in affected colonies bees detect a problem in the development of the larvae in the first hours of life and eliminate them. Accordingly, larvae fed with nectar from colonies affected with River disease or secretions of *E*. *cestri*, were observed to grow more slowly and had less mobility than larvae from other groups. For the control groups, the high larval mortality observed may be associated to the change in larval diet compared to published protocols [18]. In this study larval food had to be modified in order to add nectar (from healthy and diseased colonies).

Honey bees are capable of avoiding collecting nectars that contain toxic substances from the plants [6]. This capacity is due to a learning process in which the bees associate the particular nectar with a toxic effect [25]. However, they do not avoid collecting *E*. *cestri* secretions with toxic substances in *S*. *schottiana* trees. One possible explanation is that as toxic substances do not affect the adult bees, they cannot establish this association. It is very unlikely that bees, which obtain food from several plants, were capable two associate a carbohydrate source with larvae intoxication. Another possibility is that bees were unable to detect the toxic substances. Generalist bee species may have poor acuity for the detection of toxins in nectar because they have few gustatory receptors that can detect these compounds [26].

The trees of the genus *Sebastiania* have several substances that can be toxic to various organisms, among which the xanthoxyline and their derivates have antispasmodic activity against acetylcholine-induced contraction [27–28].

In summary, there are three lines of evidence that lead us to conclude that the secretions of *E*. *cestri* in *S*. *schottiana* trees are responsible for the death of the larvae: the observation that bees collect secretions, the fact that the period in which larvae mass mortality occurs overlaps with the presence of *E*. *cestri* and their secretions, and the abnormal death of larvae reared in the laboratory which received nectar from colonies affected with River disease or planthopper secretions. Honey bees frequently collect sugary secretions from insects without suffering any harm [29].This is the first case observed in which honeydew causes mass mortality in honey bee larvae.

Future studies should focus on identifying the substances contained in the tree sap, or that *E*. *cestri* incorporates to their secretions, that kill bee larvae and explore colony handling technique that reduce the negative impact of River disease.
